# Wnt Activation of Immortalized Brain Endothelial Cells as a Tool for Generating a Standardized Model of the Blood Brain Barrier *In Vitro*

**DOI:** 10.1371/journal.pone.0070233

**Published:** 2013-08-05

**Authors:** Roberta Paolinelli, Monica Corada, Luca Ferrarini, Kavi Devraj, Cédric Artus, Cathrin J. Czupalla, Noemi Rudini, Luigi Maddaluno, Eleanna Papa, Britta Engelhardt, Pierre Olivier Couraud, Stefan Liebner, Elisabetta Dejana

**Affiliations:** 1 IFOM-FIRC Institute of Molecular Oncology Foundation, Milan, Italy; 2 Department of Biosciences, University of Milan, Milan, Italy; 3 Institute of Neurology (Edinger Institute), Johann Wolfgang Goethe University, Frankfurt, Germany; 4 Inserm, U1016, Institut Cochin, Paris, France; 5 CNRS, UMR8104, Paris, France; 6 Université Paris Descartes, Sorbonne Paris Cité, Paris, France; 7 Theodor Kocher Institute, University of Bern, Bern, Switzerland; Washington University, United States of America

## Abstract

Reproducing the characteristics and the functional responses of the blood–brain barrier (BBB) *in vitro* represents an important task for the research community, and would be a critical biotechnological breakthrough. Pharmaceutical and biotechnology industries provide strong demand for inexpensive and easy-to-handle *in vitro* BBB models to screen novel drug candidates. Recently, it was shown that canonical Wnt signaling is responsible for the induction of the BBB properties in the neonatal brain microvasculature *in vivo*. In the present study, following on from earlier observations, we have developed a novel model of the BBB *in vitro* that may be suitable for large scale screening assays. This model is based on immortalized endothelial cell lines derived from murine and human brain, with no need for co-culture with astrocytes. To maintain the BBB endothelial cell properties, the cell lines are cultured in the presence of Wnt3a or drugs that stabilize β-catenin, or they are infected with a transcriptionally active form of β-catenin. Upon these treatments, the cell lines maintain expression of BBB-specific markers, which results in elevated transendothelial electrical resistance and reduced cell permeability. Importantly, these properties are retained for several passages in culture, and they can be reproduced and maintained in different laboratories over time. We conclude that the brain-derived endothelial cell lines that we have investigated gain their specialized characteristics upon activation of the canonical Wnt pathway. This model may be thus suitable to test the BBB permeability to chemicals or large molecular weight proteins, transmigration of inflammatory cells, treatments with cytokines, and genetic manipulation.

## Introduction

The blood brain barrier (BBB) is a highly specialized region of the vascular tree, which preserves the integrity of the nervous system by limiting the passage of harmful substances and inflammatory cells into the brain [1], [2], . The endothelial cells (EC) of the brain microvessels acquire a set of specialized functional and morphological properties through their interactions with the surrounding astrocytes and pericytes. This cross talk between the cells is vital to the functionality of the BBB. The brain EC, astrocytes and pericytes in close contact form the so-called ‘neurovascular unit’. These EC develop highly selective barrier functions due to a particularly complex inter-endothelial tight junction network and a unique set of transporters, which allow the controlled passage of nutrients and eliminate toxic substances [4].

However, the neuroprotective functions of the BBB hinder the delivery of many potentially important diagnostics and therapeutic drugs to the central nervous system. Large molecules, such as antibodies, and the majority of small molecule drugs cannot cross the BBB [5], [6]. This has dramatically delayed the progress of pharmacotherapies and immunotherapies in brain diseases. Furthermore, treatment of neurological disorders, such as multiple sclerosis, stroke and Alzheimer’s disease, is hindered by the negligible bioavailability of drugs [7], [8], [9].

The reproduction of the characteristics and the functional responses of the brain microvasculature *in vitro* represents an important task for the research community and would provide a critical biotechnological breakthrough. Thus, a crucial need for the progress in this area is a standardized *in vitro* assay that can be shared by many different laboratories to provide comparable results. Indeed, an optimal BBB system for large scale screening needs to be highly reproducible among laboratories, relatively inexpensive and easy to handle. To date, none of the available systems fits all of these criteria (for reviews see [10], [11], [12], [13]).

One major problem is that, when they are isolated from the brain microenvironment, the EC lose their specialized barrier characteristics in few days. This appears to be due to the disruption of the brain neurovascular unit, which maintains the specialized characteristics of EC only through a continuous cross talk of these cells with astrocytes, pericytes and neurons.

To avoid the loss of endothelial BBB properties, freshly isolated brain endothelium and astrocytes have been co-cultured in a two chamber cell culture system. Under these conditions, EC maintain some of their BBB properties. However, although valuable for short term analysis, this experimental model is not easily reproducible and can give variable results from one investigator to another, which prevents its use on any large scale basis (for reviews see [10], [11], [12], [13]). Therefore, further efforts are needed to replace astrocytes with a standardized medium that contains the factors necessary to maintain the BBB characteristics of brain EC.

In previously published studies [14], [15], [16], it was demonstrated that canonical Wnt signaling has a critical role in brain vascularization and in differentiation of the BBB *in vivo*. Canonical Wnt signaling is mediated by β-catenin, which, when is stabilized in the cytoplasm, can translocate to the nucleus and, through the interaction with lymphoid enhancer factor/T-cell factor (Lef/Tcf) transcription factors, modulates gene transcription.

In the present study, we have taken advantage of these early studies to investigate and define a novel BBB model in which the addition of Wnt ligands or drugs that increase canonical Wnt signaling can maintain, and even restore, the BBB properties of EC in a way similar to that of astrocyte co-cultures. The BBB properties can be maintained also by infecting the EC with a dominant active form of β-catenin that can sustain Wnt signaling in these cells [16].

Even if avoidance of the need for astrocyte co-culture is already an important standardization step, the use of freshly isolated primary brain EC cultures is still expensive, time consuming and requires operator expertise. For this reason, we used immortalized mouse brain derived microvascular EC as candidate BBB model [17], [18], and we show that this cell line provides an easy-to-handle model that maintains a number of phenotypic characteristics of the BBB EC in culture. Upon activation of the canonical Wnt signaling, these cells show increased BBB marker expression, including tight junction and adherens junction proteins. Furthermore, as cell monolayers, they show increased transendothelial electrical resistance (TEER) and reduced permeability, thus reproducing to a good extent the phenotype of the freshly isolated brain EC. In addition, similar data were obtained with the hCMEC/D3 human brain EC line [19].

In conclusion, the strategy described here provides a standardized approach to the preparation of *in vitro* models of the BBB that reproduce and maintain the specialized properties of the brain endothelium.

## Results

As a readout for the functional BBB properties, we first defined a set of genes known to be specifically expressed by freshly isolated brain EC, as listed in Table 1 (for reviews on BBB markers see [20], [21], [22]). A microfluidic card (Custom TaqMan Array; see Materials and Methods) containing the selected gene probes, which we refer to as the ‘BBB signature’, was developed and used systematically for comparisons of cell lines and culture conditions.

**Table 1:** BBB endothelial signature genes.

Gene	Probe	Description
Cdh5/VE-cadherin [50], [51]	Mm00486938_m1	Adherens junctions, Cadherin-5, vascular endothelium (VE)-cadherin
Cldn3 [52], [53], [54]	Mm00515499_s1	Tight junctional Claudin-3
Cldn5 [54], [55]	Mm00727012_s1	Tight junctional Claudin-5
Cldn12 [53], [56]	Mm01316510_m1	Tight junctional Claudin-12
Plvap/PV-1 [57], [58]	Mm00453379_m1	Plasmalemma-vesicle-associated protein
Bsg/HT-7/CD147 [59], [60]	Mm01169115_m1	Leukocyte activation antigen M6, Basigin
Slc2a1/Glut1 [61], [62]	Mm00441473_m1	Solute carrier family 2/Facilitated glucose transporter 1
Slc7a1/CAT1 [63], [64]	Mm00432019_m1	Solute carrier family 7/Cationic amino acid transporter 1
Abcg2/BCRP [65], [66]	Mm00496364_m1	ATP-binding cassette transporter G2
Abcc4/MRP4 [67], [68]	Mm01226381_m1	Multidrug-resistance-associated protein 4
Abcb1b/MDR1/P-gp [69], [70]	Mm00440736_m1	Multidrug-resistance protein 1
Lrp1/A2MR/APOER [71], [72]	Mm00464608_m1	Low-density lipoprotein receptor-related protein 1
Ager/RAGE [72], [73]	Mm01134790_g1	Receptor for advanced glycosylation end products
Gpr126 [74]	Mm01193801_m1	G-protein coupled receptor 126

**Figure 1:**
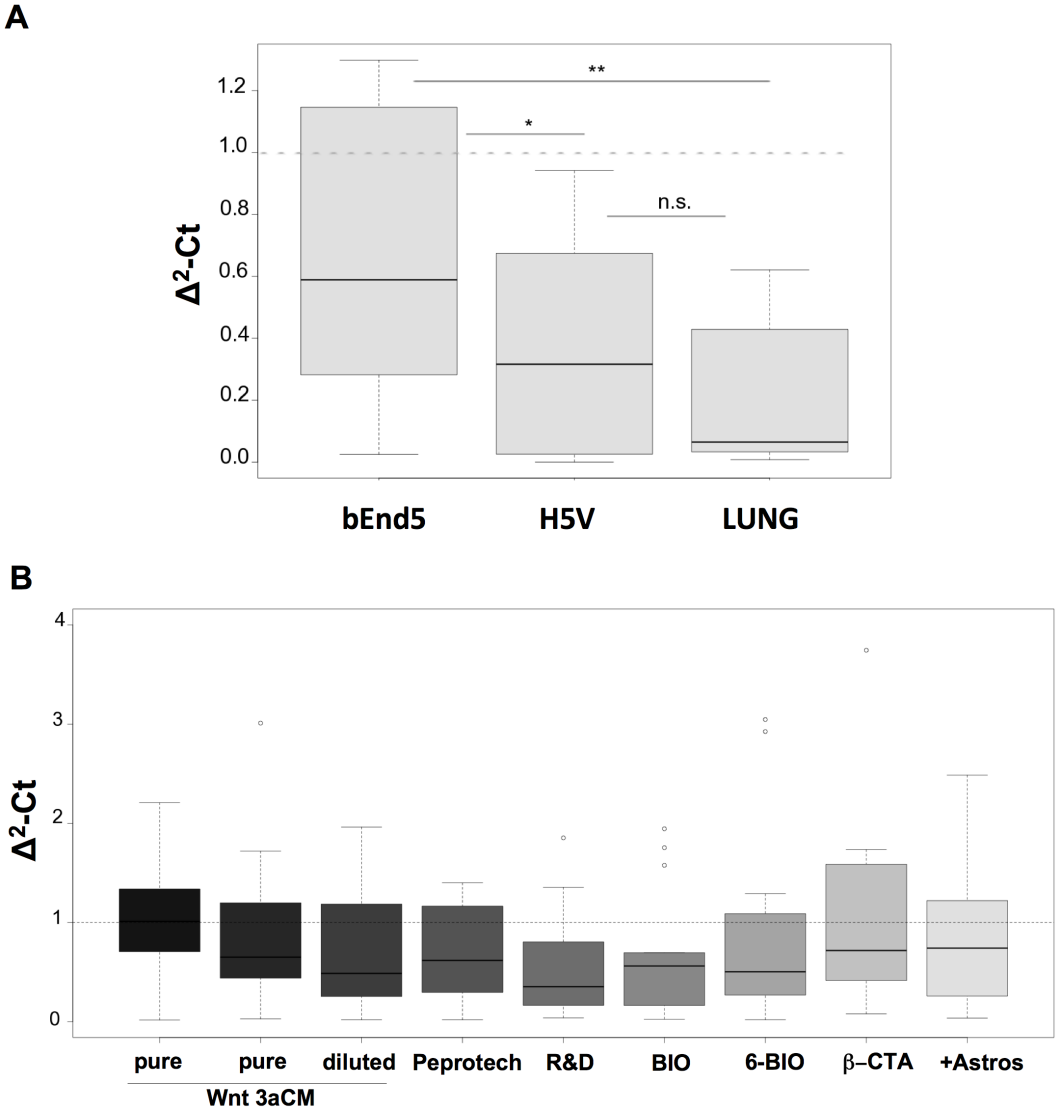
Box plots showing the genetic comparisons between immortalized mouse endothelial cell lines and primary brain microvascular endothelium as reference. **A.** Global distribution of the BBB-specific genes in the bEnd5, H5V and lung cells in comparison with MBMECs (set to 1; dotted line). A non-parametric test (i.e., Wilcox test, alpha value set at 0.05) was used to determine whether or not the differences between these cell types are significant. *p<0.1; **p<0.05; n.s., not significant. **B.** Global distribution of the BBB-specific genes in the bEnd5 cell systems, compared with MBMECs (set to 1; dotted line). Various conditions were tested: conditioned medium from Wnt3a-transfected L-cells (Wnt3aCM) undiluted (pure) or diluted 1 to 3 in growing medium; two different commercial recombinant Wnt3a preparations (100 ng/ml; Peprotech and R&D); BIO and 6-BIO (2.5 µM). Undiluted Wnt3aCM treatment was for 3 days (first boxplot from the left) or 24 hours (second boxplot from the left), as all the other cell activations. β-CTA (cells infected with LEFΔN-βCTA) and +Astros (co-culture with astrocytes). No significance differences were detected between these conditions, although a negative trend was seen for R&D Wnt3a and BIO conditions, as median values are lower than 1.

An important requirement in the development of a widely reproducible BBB model system is the use of immortalized EC lines. Using the BBB-specific gene expression card, we compared immortalized mouse EC lines derived from the heart microvascular endothelium (H5V cells) [23], the lung endothelium [24] and the brain endothelium (bEnd5 cells) [17], [18] to freshly isolated mouse brain microvascular endothelial cells (MBMECs) [25], [26] (Figure 1A). The data in the boxplot of Figure 1A are given as a quality index that summarizes the overall expression of the selected BBB EC genes as a single value, in comparison with that of MBMECs (see Materials and Methods). The precise values for the individual genes are reported in Figure S1A. Figure 1A shows that the gene expression pattern of bEnd5 cells was close to the reference MBMECs, which were here normalized to 1. Furthermore, bEnd5 cells show higher expression of tight junction proteins (Claudin-3, −5, −12) and transporters (such as Slc7a1, Abcb1b) than H5V cells and lung EC (Figure S1A). Importantly, we found that the choice of the growth medium used strongly influences the functional properties of bEnd5 cells. The cells were cultured with complete Dulbecco’s modified Eagle’s medium (DMEM) or MCDB-131 for a few passages until confluence, and the monolayers were compared at the optical microscope. Indeed, as shown in Figure S2, for bEnd5 cells, most of the BBB-associated genes were expressed at higher levels when the EC were cultured in MCDB-131 medium, as compared to DMEM (see Materials and Methods).

As well as marker gene expression, bEnd5 cells can also correctly organize their tight junction proteins (Claudin-3, −5) and adherens junction proteins (data not shown) at cell-to-cell contacts to comparable levels of MBMECs.

**Figure 2:**
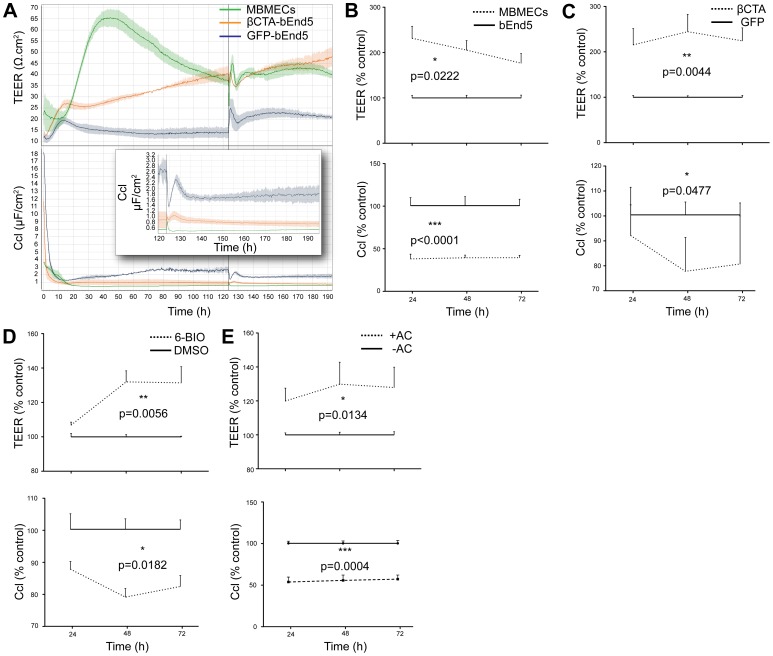
β-catenin transcriptional activity improves impedance of bEnd5 cells A–D. Transendothelial electrical resistance (TEER, top panels) and the corresponding capacitance (Ccl, bottom panels) of the endothelial monolayers at various times after they reached confluence. **A.** Representative TEER/Ccl measurement comparing MBMECs, bEnd5 and bEnd5 infected with LEFΔN-βCTA (βCTA-bEnd5), indicating that β-catenin transcriptional activation leads to increased electrical resistance in bEnd5 cells. Vertical line at 125 hours indicates media exchange and boxed insert shows magnification of the Ccl curves after the media exchange, highlighting pronounced lower values for the βCTA-bEnd5 compared to bEnd5 controls. **B.** Parental bEnd5 cells in comparison to the primary mouse MBMECs cells (n = 3). **C.** bEnd5 cells infected with lenti-LEFΔN-βCTA in comparison to the lenti-GFP control. **D.** bEnd5 cells treated with the GSK3α/β inhibitor 6-BIO (2.5 µM) in comparison to the DMSO-treated cells. **E.** bEnd5 cells co-cultured with astrocytes (+AC) in comparison to bEnd5 cell monocultures (−AC).

From these initial studies, among the immortalized cell lines tested, bEnd5 cells appeared to be a good candidate to reproduce the BBB EC characteristics. However, bEnd5 cells did not reproduce the same gene expression pattern or show the same control of cell permeability (see below) as the freshly isolated MBMECs. (Figure 2B, Figure S3).

In earlier studies [14], [15], [16], it was reported that canonical Wnt signaling is responsible for the induction of the BBB properties during brain vascularization in the embryo and in newborn mice. The canonical Wnt pathway, which is induced by Wnt3a or Wnt7a/b in the brain, acts via β-catenin stabilization in the cytosol, which favors the nuclear translocation of β-catenin and its binding to transcription factors of the Lef/Tcf family [27]. We therefore tested whether activation of bEnd5 cells by Wnt3a can restore high expression levels of the BBB EC genes. As shown in Figure 1B, various conditions were tested; i.e., conditioned medium from Wnt3a-transfected L-cells (Wnt3aCM), and two different recombinant Wnt3a preparations (Figure 1B, Peprotech and R&D [28]). bEnd5 cells cultured in undiluted Wnt3aCM significantly increased the expression of the BBB-specific genes and the application of two different recombinant preparations of Wnt3a had comparable effects, or was slightly less effective (Figure 1B). The single gene variations are shown in Figure S1B. Figure S4 shows immunofluorescence staining of bEnd5 confluent monolayers under controlCM and Wnt3aCM conditions. Interestingly, Wnt3a up-regulates the junctional expression of Claudin-3 but not of Claudin-5 in bEnd5 cells to a comparable level of MBMECs. As expected, active (unphosphorylated) β-catenin translocates to the nucleus upon Wnt3a activation, while junctional VE-cadherin is unchanged, as compared to the MBMECs (Figure S5). Altogether, these data support the concept that canonical Wnt signaling can restore and maintain the BBB EC properties both in the bEnd5 cells and the freshly isolated MBMECs.

However, although these data are encouraging, these observations are still insufficient for the development of a standardized BBB model that can be reproducibly used for large scale screening assays in different laboratories. The conditioned medium from the Wnt3a transfected cells still needs to be titrated, and the concentration of Wnt3a might vary greatly among different preparations (see Materials and Methods). Recombinant Wnt3a preparations are also relatively expensive and cannot be used for large scale experiments.

We therefore tested whether we could substitute Wnt3a with chemicals that act by increasing canonical Wnt signaling through inhibition of GSK3α/β, which is responsible for β-catenin phosphorylation and degradation [29], [30], [31]. To this end, we compared four different GSK3β inhibitors: lithium chloride (LiCl) [32], SB216763 [33], and (2′Z, 3′E)-6-Bromo-indirubin-3′-oxime (BIO) and its acetoxime analog (2′Z, 3′E)-6-Bromo-indirubin-3′-acetoxime (BIO-Acetoxime, 6-BIO) [34], [35]. LiCl and SB216763 inhibit β-catenin phosphorylation and degradation in the proteasome, while BIO and 6-BIO are cell-permeant compounds that are selective and reversible ATP-competitive inhibitors of GSK3α/β [34], [35], [36]. Of note, 6-BIO has greater selectivity for GSK3α/β and, importantly, is less cytotoxic at higher concentrations than BIO (see Merck’s specifications).

As shown in Figure S6, BIO and 6-BIO treatment increased BBB-specific gene expression more efficiently than LiCl and SB216763. BIO and 6-BIO treatments were also more comparable to Wnt3a treatment (Figure S1B), although they showed lower effects. The pie diagram in Figure S6 indicates that the BIO-activated and 6-BIO-activated bEnd5 monolayers have the highest expression levels under each condition, when expressed as the percentages of genes. The comparison of BIO, 6-BIO, Wnt3aCM and recombinant preparations of Wnt3a showed that, under all conditions, expression of the BBB endothelial cell specific genes was increased (Figure 1B).

To further standardize and simplify the procedure, we infected bEnd5 cells with a lentiviral construct that expresses a fusion protein containing the transactivation domain of β-catenin and a truncated form of the LEF-1 protein (LEFΔN-βCTA). This chimera cannot associate to cadherins, but retains its transcriptional activity [37]. In other words, this condition reproduces constant cell activation by canonical Wnt signaling.

As shown in Figure 1B and in Figure S1B, the LEFΔN-βCTA bEnd5 cells (+β-CTA) showed a level of expression of the BBB endothelial cell-specific genes that was comparable to the MBMECs and to bEnd5 cells treated with the Wnt3aCM. This LEFΔN-βCTA infection was also more effective than the BIO and 6-BIO treatments (Figure 1B). As with the MBMECs, immunofluorescence analysis confirmed the up-regulation of junctional Claudin-3 in these LEFΔN-βCTA bEnd5 cell monolayers, similar to that seen for Wnt3a and BIO activation (+β-CTA, Figure S4).

**Figure 3:**
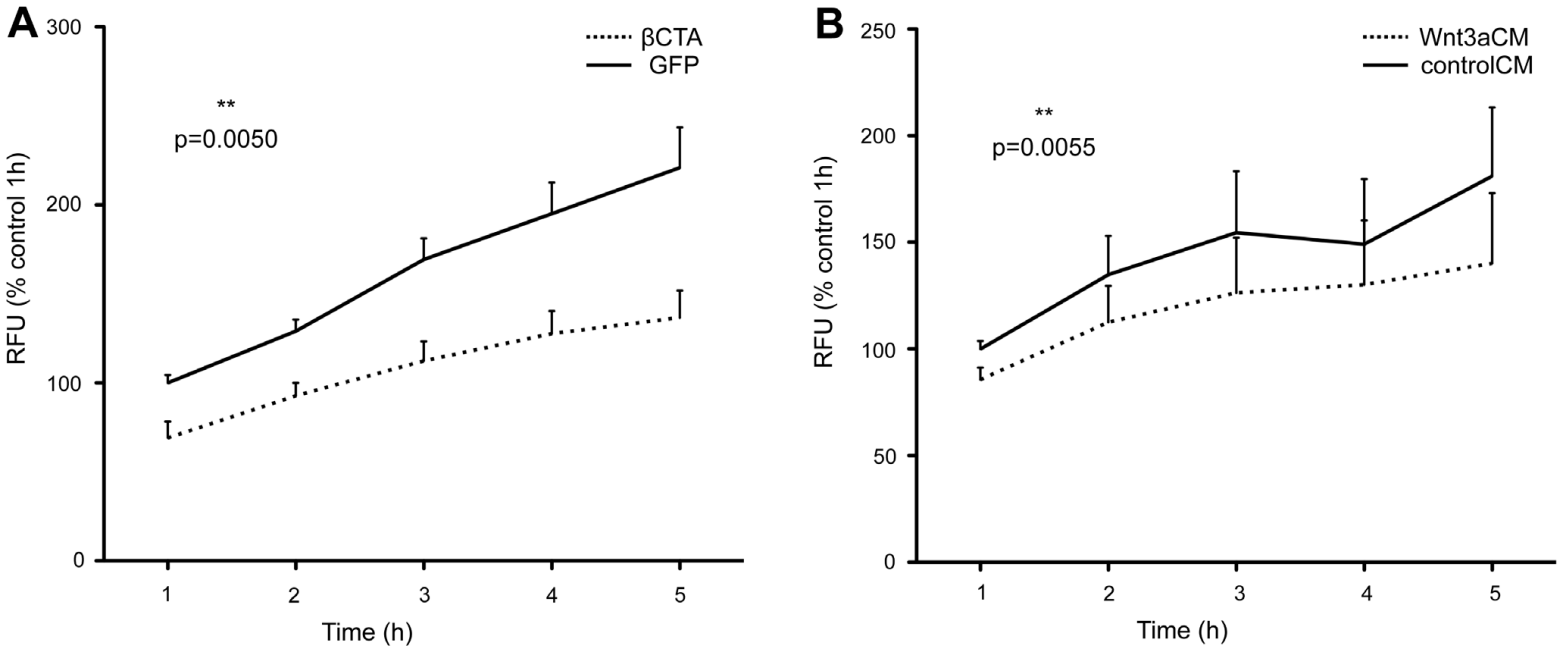
β-catenin transcriptional activity reduces dextran permeability of bEnd5 cells. **A,B.** Endothelial monolayer permeability to FITC-labeled 38-kDa dextran, was measured as percentage (%) of relative fluorescence units (RFUs). **A.** bEnd5 cells infected with LEFΔN-βCTA in comparison to GFP as control. **B.** bEnd5 cells treated with Wnt3a conditioned medium (Wnt3aCM) in comparison to the control medium (controlCM). p values were obtained by a 2-tailed paired t-test (Graphpad Prism 5.0), using values from n = 3 independent experiments, and pairing for time points.

An optimal BBB EC model must also show high TEER and restrictive paracellular permeability. Therefore, we performed studies to test these parameters on the bEnd5 cell monolayers by comparing the different conditions described above. We measured the impedance continuously over a period of ∼190 hours for MBMECs and bEnd5 transduced with or without LefΔN-βCTA (Figure 2A). MBMECs showed the absolute highest TEER of ∼65Ω.cm^2^, ∼43 hours after plating and subsequent attachment, indicated by the lowest Ccl values. But importantly, also bEnd5 cells transduced with LefΔN-βCTA (βCTA-bEnd5) exhibited a pronounced increase in TEER over the GFP controls. It is worth noting that at late times (125 hours) after media exchange, βCTA-bEnd5 recovered more robustly than the MBMECs, suggesting that βCTA-bEnd5 are suitable for long-term cultures. In order to statistically analyse TEER data we show values at 24, 48 and 72 hours for the different conditions after the EC monolayers had reached confluence as % of control (Figure 2B,C) (see Materials and Methods). The monolayers of parental bEnd5 cells showed significantly lower barrier properties in comparison to the primary MBMECs, as evidenced by the higher TEER and lower capacitance (Ccl, as indication for an adequate intact cell culture barrier) in the MBMECs (Figure 2B, Figure 3S). The monolayers of βCTA-bEnd5 boosted the barrier properties, as indicated by an increased TEER when compared to control cells (GFP infected bEnd5, GFP-bEnd5) and a concomitant reduction in the Ccl (Figure 2C, Figure 3S), bringing these values closer to those of the primary MBMECs. Monolayers of bEnd5 cells treated with the GSK3α/β inhibitor 6-BIO showed significant effects in terms of improved TEER and decreased Ccl, as compared to the DMSO-treated control (Figure 2D). Importantly, when bEnd5 cells were co-cultured with astrocytes (Figure 2E, +AC), the absolute values of TEER were close to those of cells treated with BIO but lower than those of MBMECs or βCTA-bEnd5 (Figure 2E, −AC).

We then measured the permeability of endothelial monolayers to 38kDa FITC-dextran, reported as relative fluorescence units (RFUs) (Figure 3A,B). Monolayers of βCTA-bEnd5 showed a lower dextran flux at all times measured, as compared with the control GFP-infected cell line (Figure 3A). Furthermore, monolayers of bEnd5 cells treated with the Wnt3aCM showed lower values for this dextran flux, as compared to the control (Figure 3B). Notably, parental- and GFP-infected bEnd5 monolayers did not differ significantly in impedance measurements (data not shown).

Taken together, our data show that the βCTA-bEnd5 cell line constitutes a reproducible model system of BBB. As this model is based on an immortalized cell line, it can be maintained in culture for extended times and numbers of passages. The culture conditions require a standard medium, and the parameters reported above have been reproduced over two years in the three separate laboratories that have contributed to this study. The functional parameters and BBB EC gene expression is comparable to freshly isolated MBMECs and to co-culture of the parental cell line with astrocytes.

The βCTA-bEnd5 cell system appears to be particularly suited for large scale BBB EC screening assays, as these cells do not need additional expensive reagents (e.g., Wnt recombinant proteins) or complex co-culture systems.

**Figure 4:**
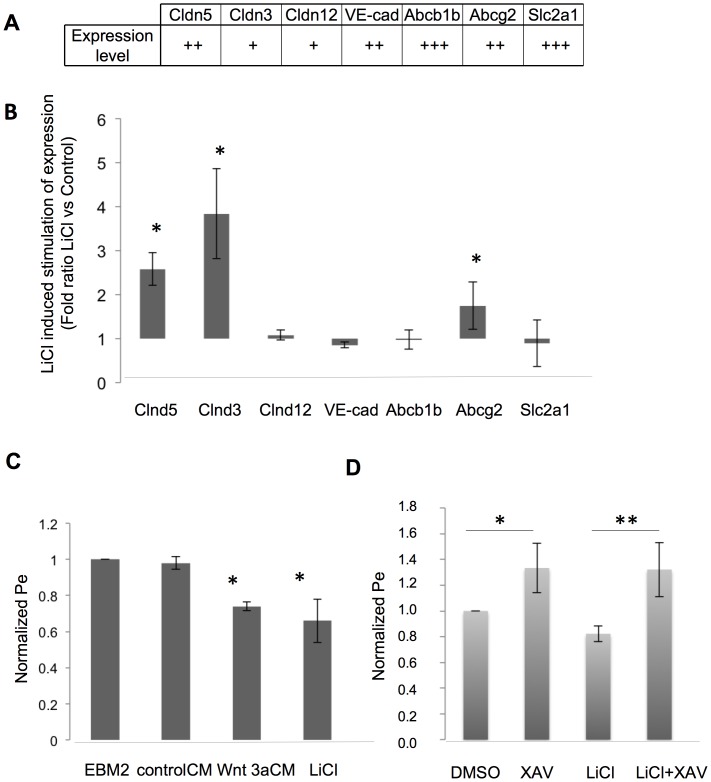
LiCl treatment improves the BBB-specific phenotype of hCMEC/D3 cells. **A.** Basal mRNA expression of the BBB endothelial cell-related genes in hCMEC/D3 cells (as indicated; +++: 20<Δ^2^-Ct<25; ++: 25<Δ^2^-Ct<30; +: 30<Δ^2^-Ct<35). (For details see statistical analysis paragraph in Materials and Methods). **B.** qRT-PCR analysis from hCMEC/D3 cells treated with 10 mM LiCl compared with untreated cells. The RNA level obtained from untreated cells was set to 1 and the ratio LiCl treated versus control is shown for each gene. * p<0.05. Cldn, Claudins; VE-cad, VE-cadherin; Abcb1b, multidrug resistance protein 1; Abcg2, ATP-binding cassette transporter G2; Slc2a1, Solute carrier family 2 (facilitated glucose transporter) 1. **C.** hCMEC/D3 cell permeability (Pe) to Lucifer Yellow. Cells were untreated (EBM2) or treated with controlCM, 50% Wnt 3aCM or 10 mM LiCl (Pe values normalized to EBM2 Pe = 1.7×10^−3^ cm/min). **D.** hCMEC/D3 cell permeability (Pe) to Lucifer Yellow. Cells were incubated with DMSO as control or 20 µM XAV939 (XAV), 10 mM LiCl, or 10 mM LiCl plus 20 µM XAV939 (LiCl+XAV; Pe values normalized to DMSO Pe = 1.65×10^−3^ cm/min). All cell treatments were performed for 6 days.

To test whether we can extend these observations to the hCMEC/D3 immortalized human brain microvascular EC line [19], we activated these cells with LiCl to stabilize β-catenin, and checked for the expression of some of the BBB endothelial cell related genes (Figure 4A,B). This treatment induced the up-regulation of the tight junction proteins Claudin-3 and Claudin-5 and the transporter Abcg2, while the other selected markers were non-significantly modified likely because they were already rather high without cell activation by LiCl (Figure 4A,B). LiCl and Wnt3aCM reduced permeability of the hCMEC/D3 cells to Lucifer Yellow (Figure 4C, Pe). In contrast the β-catenin antagonist XAV939, an Axin 2 stabilizer [38], increased permeability alone and in combination with LiCl (Figure 4D).

We conclude from these data that activation of canonical Wnt signaling may be a general strategy to optimize *in vitro* models of the BBB of various species.

## Discussion

In the present study, we propose a novel method to optimize *in vitro* BBB model systems. The method is based on activation of the canonical Wnt/β-catenin pathway of immortalized brain microvascular EC lines of mouse (bEnd5) and human origin (hCMEC/D3).

Previously, we and others have reported that canonical Wnt signaling directs brain angiogenesis during embryo development, and at a later step, induces BBB differentiation [14], [15], [16]. The continuous cross talk of EC and astrocytes is necessary to maintain the functional properties of the BBB. When brain EC are isolated and cultured in the absence of astrocytes, they rapidly lose their phenotypic properties. So, as astrocytes produce different members of the Wnt family, we sought to reconstitute the effects of the co-culture by addition of Wnt ligands or drugs that reproduce the Wnt effects. Indeed, we report here that such an approach can maintain the BBB characteristics of the EC for long periods of time and through multiple passages in culture, thus establishing an easy to handle and reproducible model. The readout for the properties of the BBB used in our study was the pattern of the specific gene expression and the control of monolayer permeability.

As further standardization of the response, and to avoid the addition of expensive reagents, we transfected the bEnd5 murine brain cell line with a mutant of β-catenin that cannot be degraded and maintains high β-catenin transcriptional activity (LEFΔN-βCTA). The LEFΔN-βCTA bEnd5 cell line reproduced, to a good extent, the characteristics of freshly isolated MBMECs. Furthermore, the LEFΔN-βCTA construct has transcriptional activity only and lacks interactions with cadherins at junctions. This excludes the possibility that our results are due to increased β-catenin levels at adherens junctions.

The hCMEC/D3 immortalized human brain EC line [19] similarly responded to canonical Wnt activation by the up-regulation of BBB endothelial cell specific gene expression and reduced monolayer permeability. Although additional experiments are required to further optimize these experimental settings, these data strongly suggest that the approach described in the present study can be extended to other types of brain EC lines, including human cells.

Others have developed experimental systems using human brain EC. Bernas and colleagues [39] described a method for the generation of primary cultures of human brain microvascular EC derived from temporal tissue microvessels, with the limitation of individual sample variability and restricted availability (human brain samples are obtainable only after informed consensus and consideration by institutional review boards) [39]. This essentially prevents the use of this model for large scale screening assays.

A recent study described the derivation of EC from human pluripotent stem cells [40]. Through this procedure, the authors were able to reproduce the characteristics of the BBB using a co-differentiation procedure of EC with neural cells. This assay is of great value for studies of the differentiation steps of the BBB EC, but it is not suitable for drug screening for the complexity of the experimental procedure.

In conclusion, activation of the Wnt pathway is a relatively easy and versatile tool to establish and maintain the BBB properties of different types of EC *in vitro*. Although this method still has limitations, such as the lack of exposure to hemodynamic forces, the use of immortalized cell lines under standardized culture conditions makes this assay reproducible across different laboratories and suitable for large scale screening assays.

## Materials and Methods

### Animals

For MBMECs preparation C57Bl6 wild-type 2 months-old mice were used; for astrocytes preparation 4 days old pups were used (Charles River Laboratories).

### Animal Statement

Mice were housed according to the guidelines set out in Commission Recommendation 2007/526/EC - June 18, 2007 on guidelines for the accommodation and care of animals used for experimental and other scientific purposes. At the end of the experiment, mice were euthanized by inhalation of high concentrations of CO_2_. Procedures involving animals and their care conformed to institutional guidelines in compliance with national law and policies (4D.L.N.116, G.U., supplement 40, 18-2-1992) and approved by the Italian Ministry of Health. All efforts were made to minimize the number of animals used and their suffering.

EC Isolation and CultureMouse brain microvascular fragments were processed as previously described [25], [26]. Capillary fragments were seeded on Collagen I (BD Biosciences) coated wells and cultured in DMEM (Life Technologies)+20% fetal calf serum (FCS; Hyclone) supplemented with 100 µg/ml heparin (Sigma) and 5 µg/ml ECGS (EC Growth Supplement, homemade from calf brain [24]). After 2 days of puromycin selection (4 µg/ml) cells were exposed for 4–5 days to conditioned medium of L-cells producing Wnt3a as described [16] (Wnt3aCM, controlCM).

Immortalized mouse EC were isolated and cultured as previously described [24]. Briefly, lung EC were grown on 0.1% gelatin-coated plates in MCDB-131 medium (Life Technologies)+20% FCS, supplemented with 100 µg/ml heparin, and 5 µg/ml ECGS [24].H5V cell line was originally isolated from the heart microcirculation of E15 fetus [23], [24] and the culture medium was DMEM+10% FCS, 100 µg/ml heparin and 5 µg/ml ECGS.

Immortalized brain derived EC, bEnd5 cells [17], [18], were grown on 0.1% gelatin or 5 µg/ml fibronectin (Sigma)-coated plates, from passage 18 to 25, in MCDB-131 supplemented with 20% FCS, 100 µg/ml Heparin and 5 µg/ml ECGS. In some experiments, when indicated, bEnd5 were grown in DMEM supplemented with 10% FCS, 1% nonessential amino acids, as described in the literature [41].

Immortalized cerebral microvascular EC of human origin, hCMEC/D3 [19], were plated on the top of 12-well transwell insert, 0.4 µm pore size (Corning), coated with 150 µg/ml rat tail collagen type I (R&D Systems). The culture medium was EBM-2 (Lonza) supplemented with 5% FBS (PAA Laboratories GmbH) 10 mM HEPES (PAA Laboratories), 1% chemically defined lipid concentrate (Invitrogen), 1.4 µM hydrocortisone, 5 µg/ml ascorbic acid, and 1 ng/ml bFGF (Sigma). When indicated LiCl, Wnt3aCM at 50%, XAV939 (Sigma) and DMSO as a control, were added to the medium for 6 days to activate the cell monolayers.Culture of Murine Astrocytes

Cerebral cortices from 4 days old mouse pups were isolated in HBSS (with Ca^++^ and Mg^++^; Life Technologies) and digested with 0.05% trypsin/EDTA (Sigma) and 50 µg/ml DNase I (Roche). Cortical isolates were seeded on 0.001% poly-L-lysine-coated 25-ml flasks, and maintained in DMEM with 10% FCS. The primary neuroglia was determined to be >95% pure by glial fibrillary acidic protein immunoreactivity and fluorescence-activated cell sorting analysis (FACS) (data not shown).

### Reagents and Antibodies

Recombinant Wnt3a (100 ng/ml; R&D systems or Peprotech) or phosphate-buffered saline (PBS) supplemented with 0.1% FCS as control; LiCl (10 mM, Sigma), SB216763 (10 µM, Sigma), BIO (2.5 µM, Merck) and its acetoxime analog 6-BIO (2.5 µM, Merck), XAV939 (20 µM, Sigma) or DMSO as control. Wnt3aCM [28] was obtained by culturing L-cells transfected with a murine Wnt3a-expressing vector (with the parental cell line as control) (ATCC#CRL-2647 and #CRL-2648) [42]. The controlCM and Wnt3aCM were added to the cell monolayers either undiluted or 1∶3 diluted in growing medium.

Wnt3aCM activity, measured as Axin2 induction, was compared to commercially available recombinant Wnt3a (R&D systems [28] and Peprotech). A dose response for recombinant Wnt3a (25, 50, 100, 150 or 200 ng/ml for 24 hours) was tested on the bEnd5 cell line. The activity of undiluted Wnt3aCM corresponds to a concentration of around 100 ng/ml of the purified one confirming what previously reported [42].The following primary antibodies were used in immunofluorescence: mouse anti-Claudin-5 (Invitrogen), rabbit anti-Claudin-3 (Invitrogen), rat anti-VE-cadherin (clone BV13, e-Bioscience) [43], active-β-catenin antibody (8E7, Millipore). For secondary detection, species-specific Alexa Fluor-coupled secondary antibodies were used.

### Co-culture Experiments

To create a ‘close-contact’ co-culture system, astrocytes (4.5×10^4^ cells) were seeded on the basal side of 24-well transwell insert (0.001% PLL-coated polyester membrane clear, pore size 0.4 µm, Corning). Upon the astrocytes adhesion, 2.0 ×10^5^ bEnd5 cells were seeded on the apical surface of the same insert coated with fibronectin (5 µg/ml).

Finally the inserts with bEnd5 and astrocytes were transferred into the outer chamber and the cells were grown in DMEM with 10% FCS. After 3 days of co-culture, the cell monolayers were either assayed for TEER and Ccl measurements or lysed for RNA extraction.

### RNA Preparation and qRT-PCR

Total RNA was extracted with RNeasy Kit (QIAGEN) and 1 µg was reverse transcribed with random hexamers (High Capacity cDNA Archive Kit, Applied Biosystems), in accordance with the manufacturer's instructions and as described previously [25]. Micro fluidic card (qPCR cards, Applied Biosystems) was created for array profiling of the transcripts of the “BBB signature” genes. cDNA was amplified with the TaqMan Gene Expression Assay (Applied Biosystems) in an ABI/Prism 7900 HT thermocycler.

Total RNA from hCMEC/D3 cells was prepared as previously described [19]. 1 µg was reverse transcribed in a reaction mixture containing 500 µM of each dNTP, 10 mM DTT, 0.15 µg/µl random hexamers primers (Amersham Biosciences), 20 U RNaseOUT (Invitrogen) and 100 U superscript II RNase reverse transcriptase (Invitrogen). Quantitative RT-PCR SYBR GREEN fluorescein mix (Roche) was used following the manifacturer’s instructions. Specific primers for each gene were designed using the OLIGO 6.42 software (MedProbe). The primer sequences used are shown in Table S1.

### Lentivirus Infection

The lentiviral constructs (Lenti-GFP and Lenti-LEFΔN-βCTA), the viral preparation and infections were previously described [16], [44].

### Immunofluorescence Microscopy

The cells cultured in 35 mm diameter Petri dishes or 8-well chamber slides (Ibidi) were fixed with either 4% paraformaldehyde for 20 min at room temperature (RT), or with ice-cold methanol at −20°C for 5 min. Blocking (30 min), primary antibodies (1 hour, RT) and secondary antibodies (45 min, RT) were either in PBS with 2.5% skimmed milk and 0.3% TritonX-100 or in 2% BSA/PBS [45]. Immunofluorescence microscopy was performed either with Leica TCS AOBS or Nikon C1-si confocal microscopy. The images produced were processed by Adobe Photoshop and ImageJ software.

### Impedance Measurements

Impedance measurements were performed with a cellZscope device (nanoAnalytics), according to manufacturer specifications, using the default frequency protocol, starting at 1 Hz and ending at 100 kHz, as previously described [46]. The complex impedance (Z) of the EC monolayer is a function of its TEER and its capacitance (Ccl). An iteration algorithm was applied to fit the measured Z values to a parametric function and to calculate TEER and capacitance Ccl, assuming a parallel circuit of TEER and capacitance to describe the EC monolayer [47]. The preparation of EC monolayers for electrical measurements was similar to the permeability assay set-up (see below), with a few modifications. Briefly, 10^5^ cells/cm^2^ (primary cells from passage 0) or 1.5×10^5^ cells/cm^2^ (as bEnd5 cells at passage 20–25) were plated on 1.0 µm 24-well polyethylene terephthalate transwell inserts (Greiner Bio-One) coated with 5 µg/cm^2^ fibronectin. The inserts were transferred to the cellZscope instrument and measurements were commenced. The medium was changed every 3 days. All of the treatments started after the monolayer reached confluency, as judged from a plateau in the TEER and Ccl values, which were typically attained at 72 hours after seeding. For all of the treatments, the supplemented medium was added to both apical and basal chambers.

### Permeability Assay

Paracellular permeability through the bEnd5 monolayer was measured as previously described [44]. Briefly, EC were cultured to confluency on glutaraldehyde-cross-linked gelatin Transwell units (0.4 µm pore, Corning Costar) for 5 days. 1 mg/ml of 38-kDa FITC-dextran (Sigma) was added in the apical compartment.

At different times, 50 µl samples were taken from the lower compartment to measure fluorescence (492/520 nm, absorption/emission wavelengths) using a Wallac Victor3 1420 multilabel fluorometer counter (Perkin Elmer).

Paracellular permeability through the hCMEC/D3 monolayer was measured as already mentioned [19]. The cells were seeded onto culture inserts for 6 days. At the time of analysis, the culture medium was replaced with transport buffer (10 mM HEPES, 1 mM sodium pyruvate and HBSS, Invitrogen) in the lower chamber. In the upper chamber, 50 µM Lucifer Yellow salt in transport buffer was added. To quantify the tracer passage, the abluminal compartment was analyzed at each time points using a fluorometer (Fusion, Packard Bioscience Company) at the excitation and emission wavelengths of 425 nm and 538 nm, respectively. Permeability calculations were performed using the clearance principle, as described by Siflinger-Birnboim and colleagues [48]. The data are expressed as the permeability coefficient (Pe) in 10^−3^cm/min or as the percentage of permeability normalized to the permeability coefficient for the control conditions of untreated cells.

### Statistical Analysis

#### Comparison of candidate cell lines

A genetic comparison between a given cell line and a reference cell (e.g., freshly isolated brain primary cells) starts with the development of an RT-PCR assay for a specific set of genes of interest (Table 1). As a result, normalized delta-delta Ct values (Δ^2^-Ct, one for each gene) expressing the fold-change in the mRNA expression of the candidate cell line with respect to the reference cells is obtained. Furthermore, post-processing analysis of raw Ct data also provides normal standard errors and standard deviations for the Δ^2^-Ct values. The results obtained in our analysis are shown in Table S1. The normalization was performed against two housekeeping genes, 18S and GAPDH, using the normalization method described in [49].

Given two candidate cell lines, the two corresponding sets of Δ^2^-Ct can be considered (candidate cell lines against reference cell line), and a non-parametric test can be performed (i.e., Wilcox test, alpha value set at 0.05), to determine whether or not the difference between these cells is significant.

Graphically, the candidate cell lines can be compared using box-plots. These show a horizontal solid line at the half-percentile (i.e., 50%, the median) of the distribution; the bottom and top horizontal lines represent the lower and upper quartiles (25% and 75% of the distribution); whiskers, when present, extend to a maximum of ±1.5-times the inter-quartile (i.e., 75% value –25% value), with values more extreme represented by circles (outliers). For the bEnd5, H5V and lung EC comparisons, the following selected BBB genes were included in the analysis: VE-cadherin (Cdh5), Claudin-3 (Cldn3), Claudin-5 (Cldn5), Claudin-12 (Cldn12), Basigin (Bsg), Plasmalemma-vesicle-associated protein (Plvap), P-gp (Abcb1b) and Glut-1 (Slc2a1) (See Table 1).

#### Comparison of different modes of activation of bEnd5 cells

Different GSK3β inhibitors were compared following the same procedures described previously for comparisons of terrains. The expression values (Δ^2^-Ct) of the signature genes were reported as a histogram for the different conditions. Due to the heterogeneity of the results, we further evaluated the percentages of genes with the highest expression values for each condition and summarized this information using a pie diagram (see Figure S6).

#### Comparison of different culture conditions of bEnd5 cells

The two media tested in our study were compared in terms of expression of the signature genes. We report the histogram of expression values (in terms of Δ^2^-Ct), with the standard deviations linked to technical replicates. As there were heterogeneous results for the different genes, we summarized these by counting the percentage of genes that were better expressed in one medium with respect to the other. This information is illustrated in a pie diagram (see Figure S2).

## Supporting Information

Figure S1 **Genetic comparisons between immortalized mouse endothelial cell lines with primary brain microvascular endothelium set as reference. A.** Histograms of single gene distributions in the immortalized bEnd5, H5V and lung cells, in comparison to primary MBMECs (set to 1; red line). **B.** Histograms of single gene distributions in the different bEnd5 cell treatments in comparison with primary MBMECs (set to 1; red line). For all cell treatment details and abbreviations refer to the Figure legend 1B.
(TIF)

Figure S2 **Comparison of bEnd5 cell culture conditions.** Expression levels (Δ^2^-Ct) with standard deviations of the technical replicates of bEnd5 cultured in DMEM or MCDB-131 cell culture media. The pie diagram shows the percentages of genes that are better expressed under one condition with respect to the other.
(TIF)

Figure S3 **Spectra for a single well from the 3 conditions depicted in** **Figure 2A** **.** We have selected the time points 24, 48, and 72 hours of a single well for each condition that is shown in Figure 2B as % control TEER and Ccl values. The frequency protocol for obtaining the total impedance values has been described in the methods section. Briefly, the impedance values (Z) for the frequencies between 100–1000 Hz are described to primarily affect the TEER and Ccl values of endothelial cells. An increase in the height of the plateau represents an increase in TEER and an increase in the breadth of the plateau represents a decrease in Ccl.
(TIF)

Figure S4 **Wnt3a pathway activation upregulates the expression of the tight junction protein Claudin-3 in bEnd5 cells similarly to primary MBMECs.** Immunofluorescence staining for Claudin-3 and Claudin-5 in MBMECs (top-left panels) and bEnd5 cells (top-right panels) treated with Wnt 3aCM or controlCM for 24 hours. The same stainings are also performed in bEnd5 cells upon treatment with either BIO or DMSO as control (bottom-left panels) and upon the infection with lentivirus expressing LEFΔN-βCTA (+βCTA) or GFP as control (bottom-right panels). Scale bar: 20 µm
(TIF)

Figure S5 **Wnt3a pathway activation does not modify VE-cadherin junctional localization but promotes β-catenin nuclear translocation in both bEnd5 and primary MBMECs.** Immunofluorescence staining for active β-catenin and VE-cadherin both in MBMECs and bEnd5 treated with Wnt3aCM or controlCM. White arrowheads indicate the nuclear localization of active β-catenin. Scale bar: 20 µm
(TIF)

Figure S6 **Comparison of different GSK3β inhibitors on cell activation.** Expression levels (Δ^2^-Ct) of the BBB endothelialcell-specific signature genes under the four different GSK3β inhibition conditions, as indicated. The pie diagram summarizes gene heterogeneity and shows the percentages of genes that are better expressed under one condition in comparison to the others.
(TIF)

Table S1 **BBB endothelial cell-specific gene signature list for human hCMEC/D3 cells (Forward primers, 5′–3′; reverse primers, 3′–5′).**
(DOCX)

Table S2 **Normalized** Δ**^2^-Ct values for the signature genes across all of the cell lines.**
(DOCX)
