# Supplementary figures and images for: Wnt Activation of Immortalized Brain Endothelial Cells as a Tool for Generating a Standardized Model of the Blood Brain Barrier In Vitro
Source: PLoS One. 2013 Aug 5;8(8):e70233. doi: 10.1371/journal.pone.0070233 (PMC3734070; doi:10.1371/journal.pone.0070233)

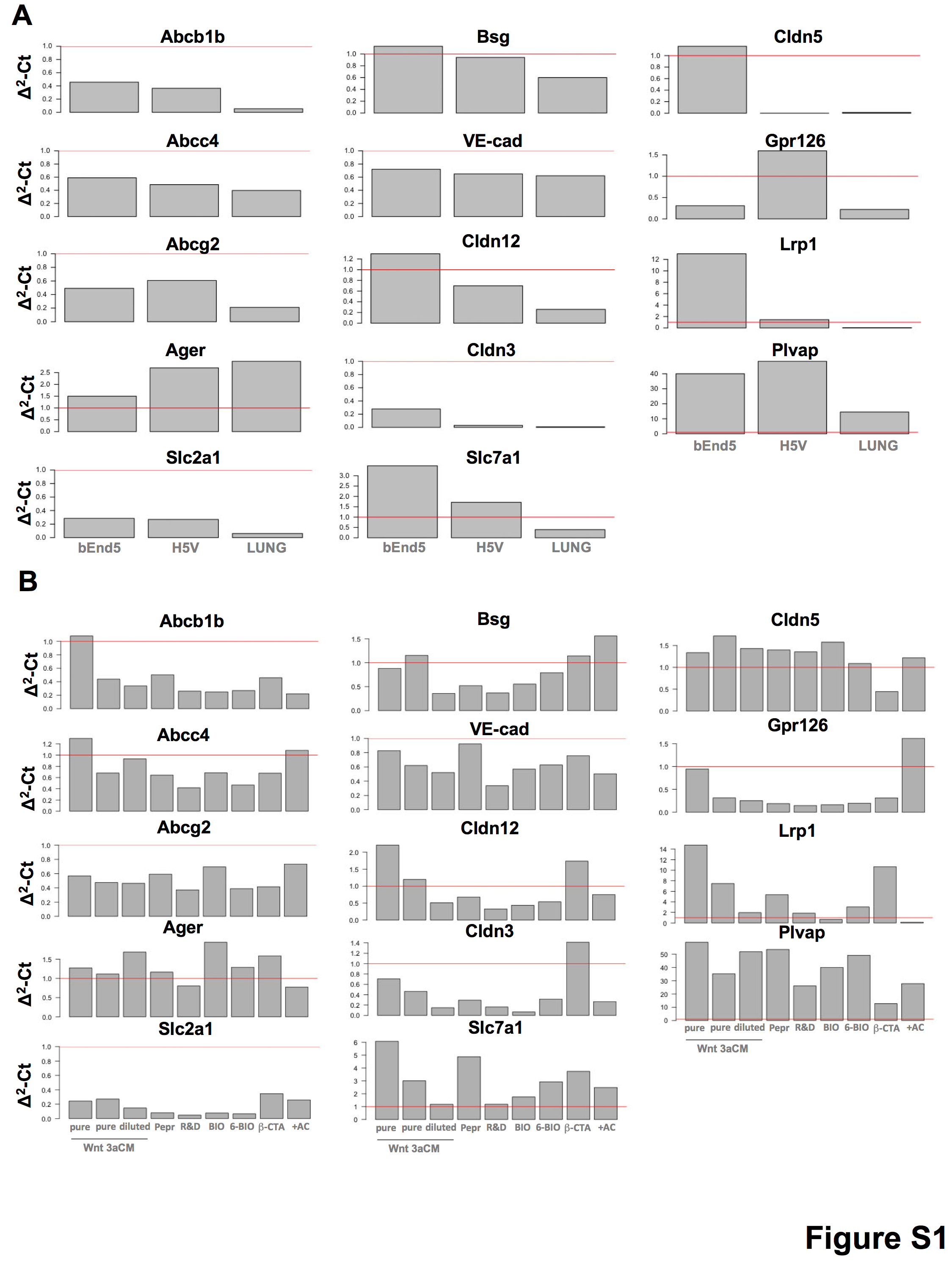

Supplement: Figure S1 — Genetic comparisons between immortalized mouse endothelial cell lines with primary brain microvascular endothelium set as reference. A. Histograms of single gene distributions in the immortalized bEnd5, H5V and lung cells, in comparison to primary MBMECs (set to 1; red line). B. Histograms of single gene distributions in the different bEnd5 cell treatments in comparison with primary MBMECs (set to 1; red line). For all cell treatment details and abbreviations refer to the Figure legend 1B. (TIF) [file pone.0070233.s001.tif]

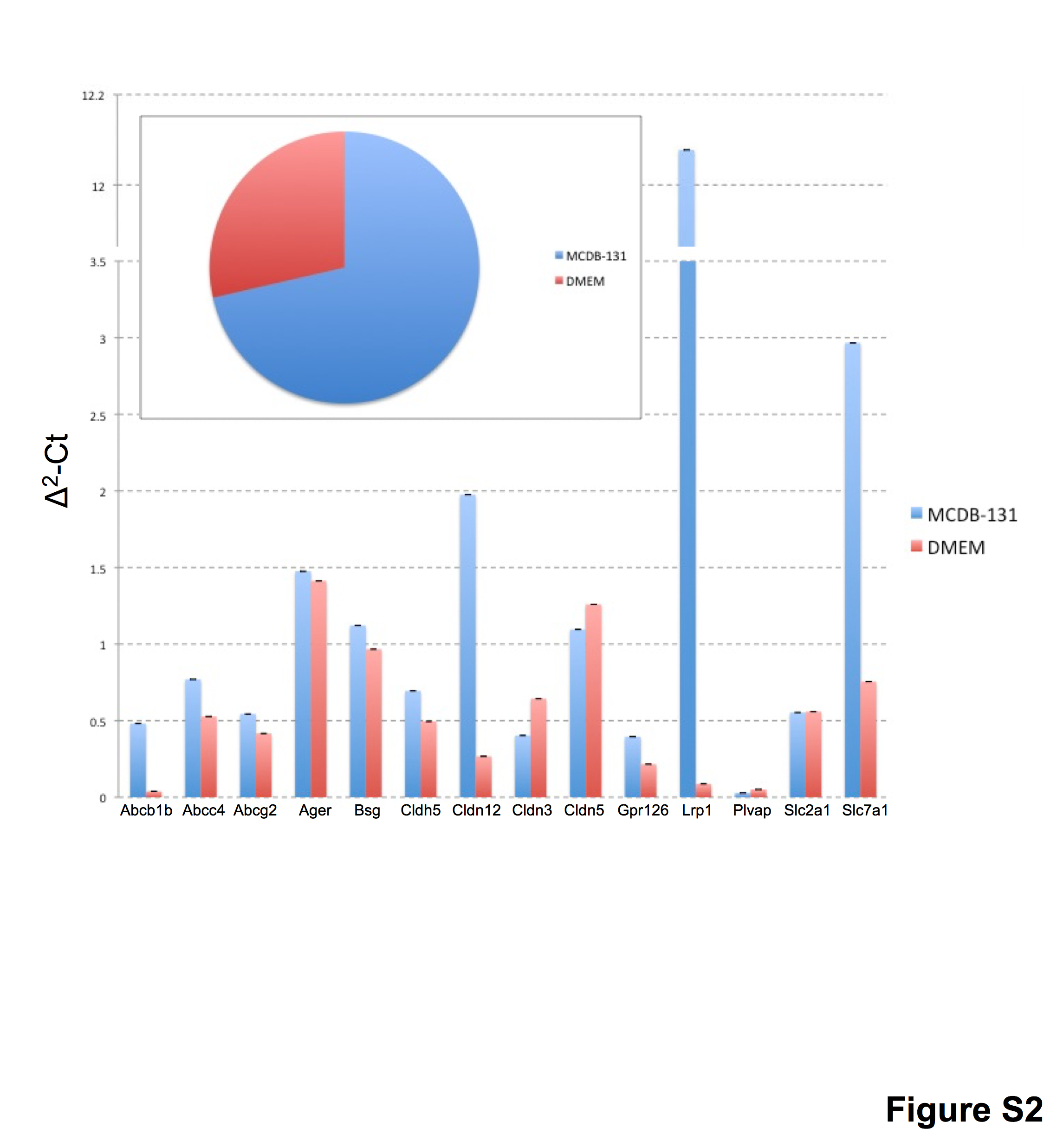

Supplement: Figure S2 — Comparison of bEnd5 cell culture conditions. Expression levels (Δ2-Ct) with standard deviations of the technical replicates of bEnd5 cultured in DMEM or MCDB-131 cell culture media. The pie diagram shows the percentages of genes that are better expressed under one condition with respect to the other. (TIF) [file pone.0070233.s002.tif]

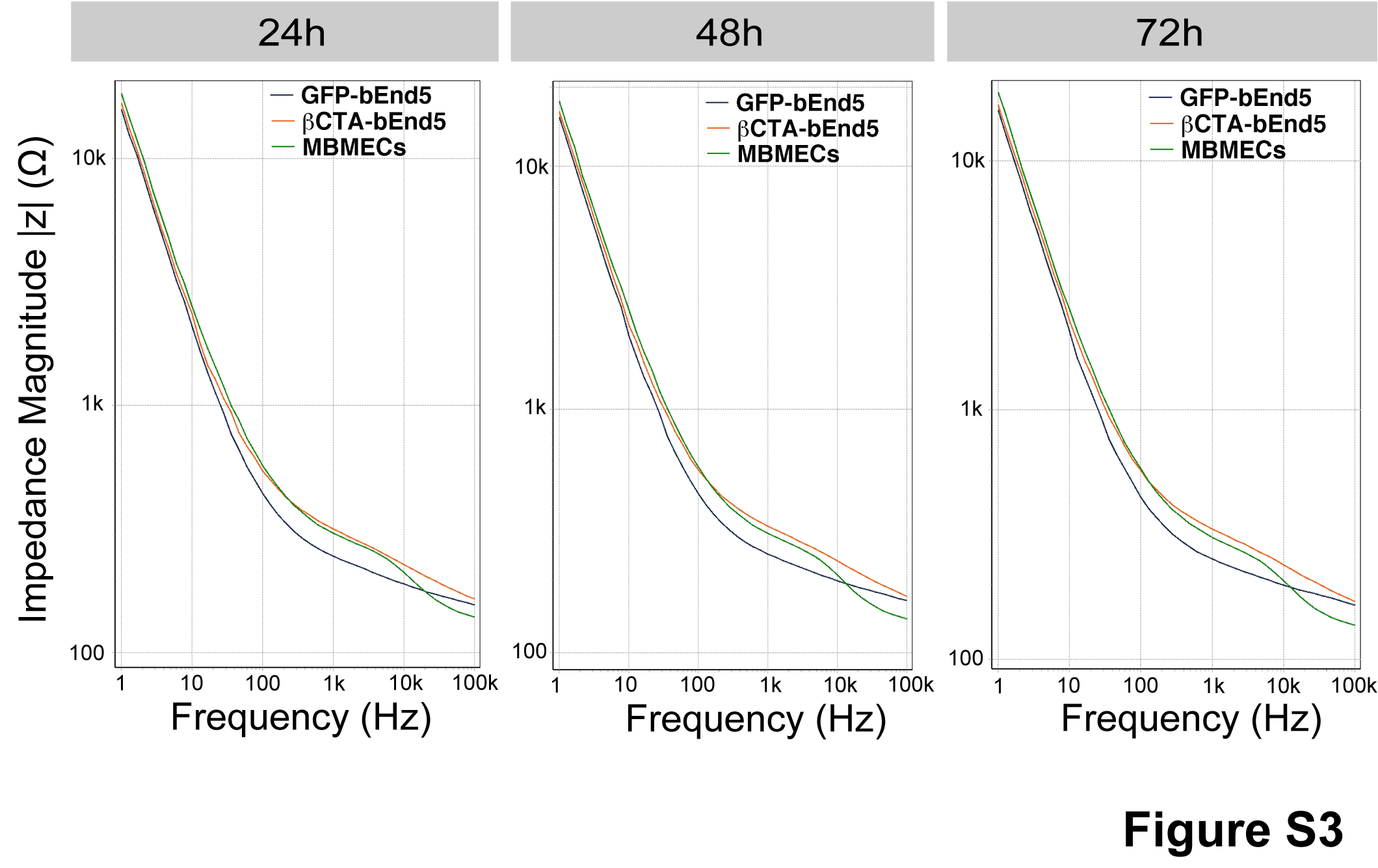

Supplement: Figure S3 — Spectra for a single well from the 3 conditions depicted in Figure 2A . We have selected the time points 24, 48, and 72 hours of a single well for each condition that is shown in Figure 2B as % control TEER and Ccl values. The frequency protocol for obtaining the total impedance values has been described in the methods section. Briefly, the impedance values (Z) for the frequencies between 100–1000 Hz are described to primarily affect the TEER and Ccl values of endothelial cells. An increase in the height of the plateau represents an increase in TEER and an increase in the breadth of the plateau represents a decrease in Ccl. (TIF) [file pone.0070233.s003.tif]

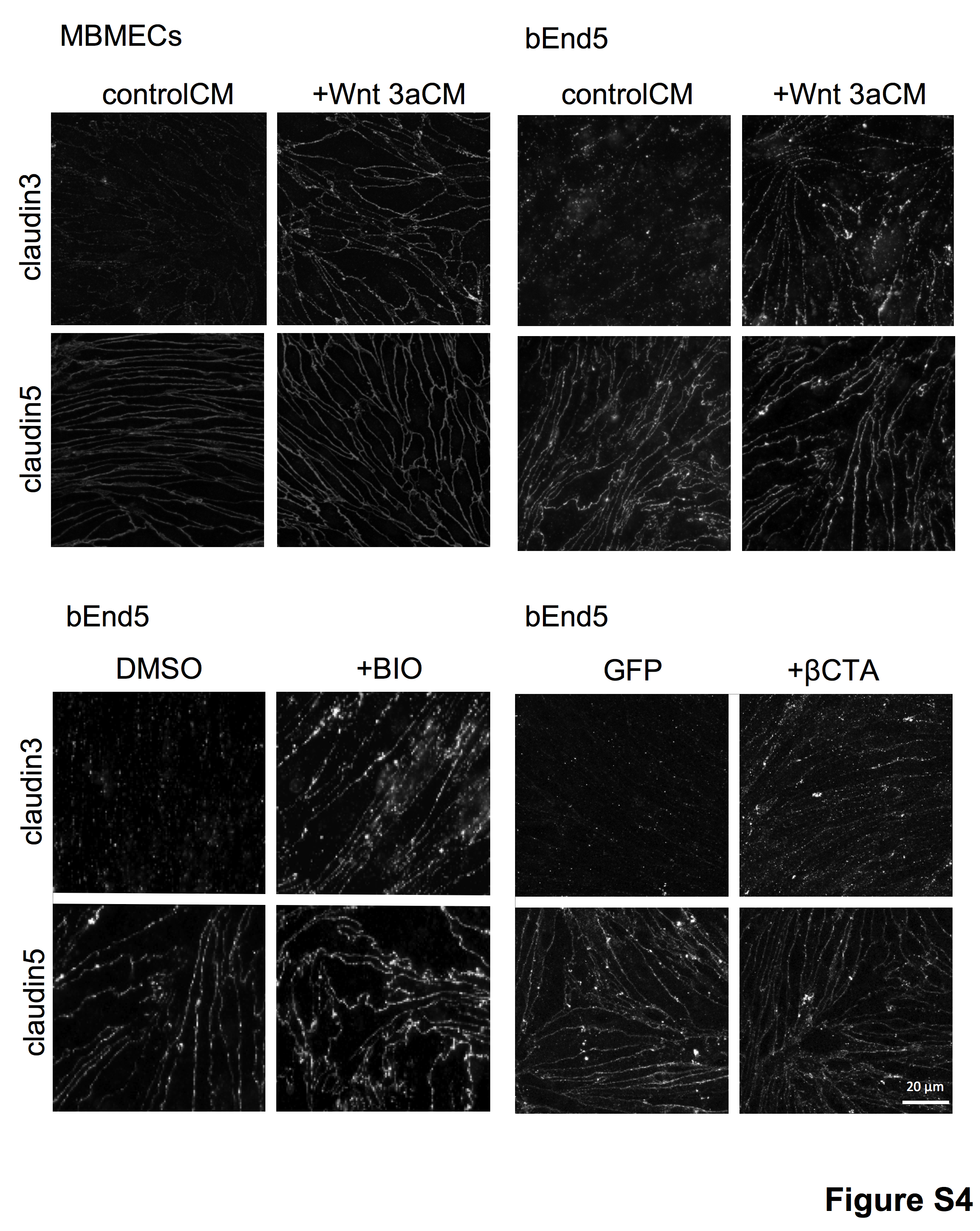

Supplement: Figure S4 — Wnt3a pathway activation upregulates the expression of the tight junction protein Claudin-3 in bEnd5 cells similarly to primary MBMECs. Immunofluorescence staining for Claudin-3 and Claudin-5 in MBMECs (top-left panels) and bEnd5 cells (top-right panels) treated with Wnt 3aCM or controlCM for 24 hours. The same stainings are also performed in bEnd5 cells upon treatment with either BIO or DMSO as control (bottom-left panels) and upon the infection with lentivirus expressing LEFΔN-βCTA (+βCTA) or GFP as control (bottom-right panels). Scale bar: 20 µm (TIF) [file pone.0070233.s004.tif]

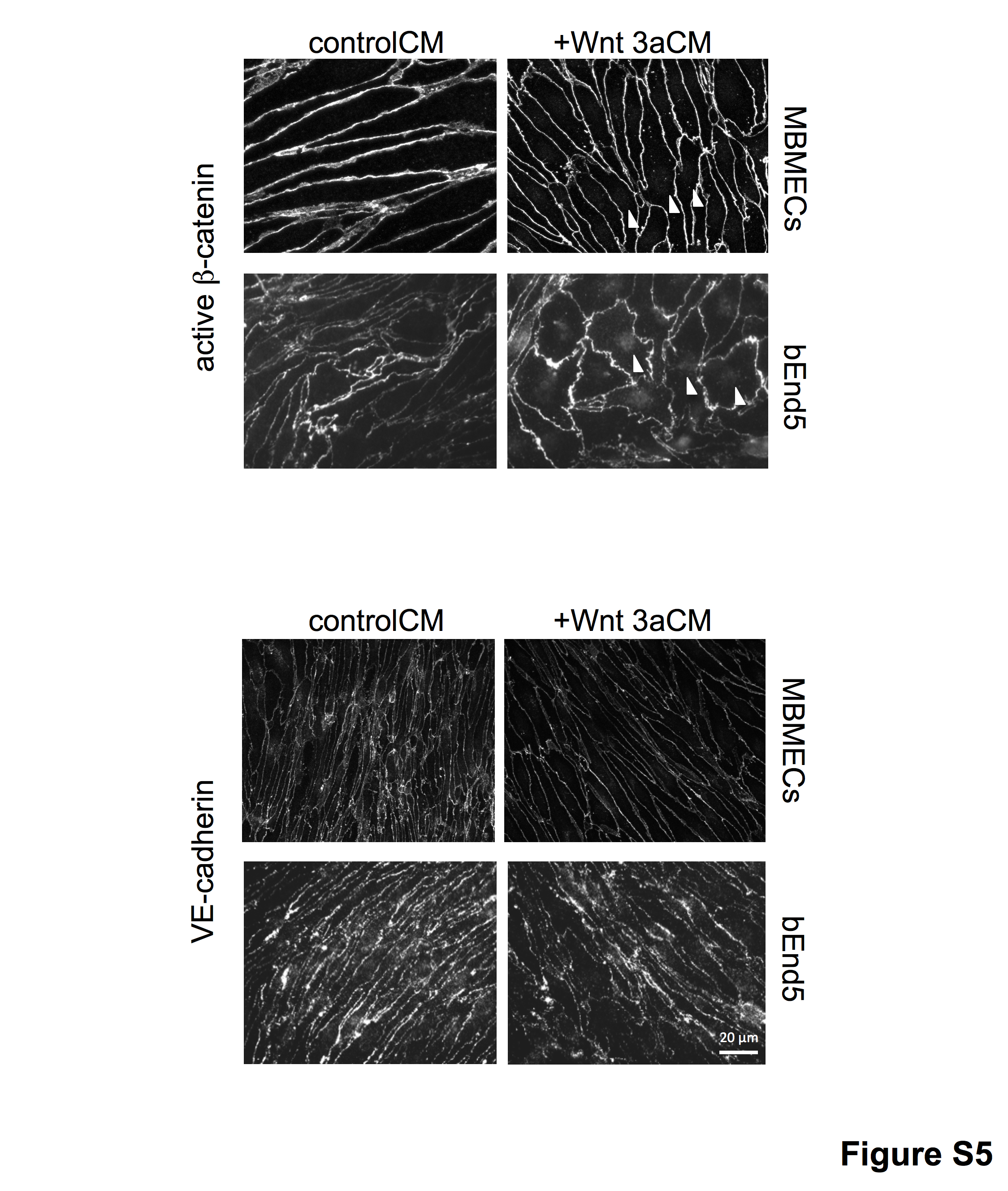

Supplement: Figure S5 — Wnt3a pathway activation does not modify VE-cadherin junctional localization but promotes β-catenin nuclear translocation in both bEnd5 and primary MBMECs. Immunofluorescence staining for active β-catenin and VE-cadherin both in MBMECs and bEnd5 treated with Wnt3aCM or controlCM. White arrowheads indicate the nuclear localization of active β-catenin. Scale bar: 20 µm (TIF) [file pone.0070233.s005.tif]

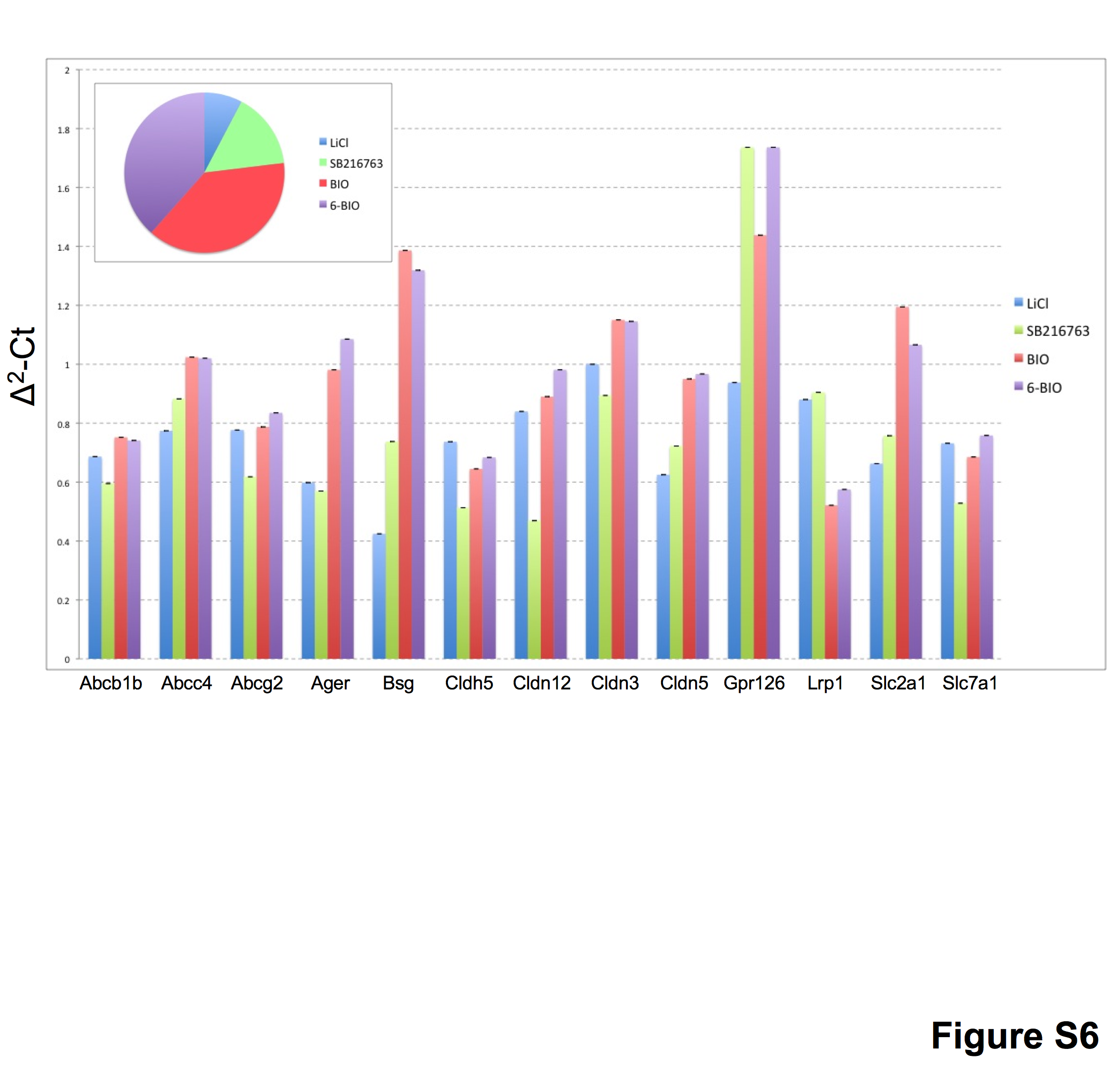

Supplement: Figure S6 — Comparison of different GSK3β inhibitors on cell activation. Expression levels (Δ2-Ct) of the BBB endothelialcell-specific signature genes under the four different GSK3β inhibition conditions, as indicated. The pie diagram summarizes gene heterogeneity and shows the percentages of genes that are better expressed under one condition in comparison to the others. (TIF) [file pone.0070233.s006.tif]
