# Supplementary material for: Wnt Activation of Immortalized Brain Endothelial Cells as a Tool for Generating a Standardized Model of the Blood Brain Barrier In Vitro
Source: PLoS One. 2013 Aug 5;8(8):e70233. doi: 10.1371/journal.pone.0070233 (PMC3734070; doi:10.1371/journal.pone.0070233)
Supplement: Table S1 — BBB endothelial cell-specific gene signature list for human hCMEC/D3 cells (Forward primers, 5′–3′; reverse primers, 3′–5′). (DOCX) [file pone.0070233.s007.docx]

**Table S1.** BBB endothelial cell-specific gene signature list for human hCMEC/D3 cells (Forward primers, 5'-3'; reverse primers, 3'-5'**)**.

| **Gene** | **Gene bank accession** | **Primer** | **Sequence** | **Length (bp)** |
| --- | --- | --- | --- | --- |
| Cldn5 | NM_001130861.1 | Forward | AGCCCCTGTGAAGATTGA | 116 |
|  |  | Reverse | TCTGGAGCCTGAGTCTCTG |  |
| Cldn3 | NM_001306.3 | Forward | CTGCTCTGCTGCTCGTGT | 143 |
|  |  | Reverse | CCTGCGTCTGTCCCTTAGA |  |
| Cldn12 | NM_012129.2 | Forward | TTTAGTAGGGGACAAGAAGTCTG | 122 |
|  |  | Reverse | AGGGTGAATGAATCAAGGTTAT |  |
| VE-cad | NM_001795 | Forward | GGACCTCAGTCATCTCTGTGA | 89 |
|  |  | Reverse | CCCCTTCAGCATTTGGTAC |  |
| Abcb1b | NM_000927 | Forward | CACCCGACTTACAGATGATG | 81 |
|  |  | Reverse | GTTGCCATTGACTGAAAGAA |  |
| Abcg2 | NM_004827 | Forward | TGACGGTGAGAGAAAACTTAC | 122 |
|  |  | Reverse | TGCCACTTTATCCAGACCT |  |
| Slc2a1 | NM_006516.2 | Forward | TGCGGGAGAAGAAGGTCA | 114 |
|  |  | Reverse | CAGCGTTGATGCCAGACA |  |
