# Supplementary material for: Wnt Activation of Immortalized Brain Endothelial Cells as a Tool for Generating a Standardized Model of the Blood Brain Barrier In Vitro
Source: PLoS One. 2013 Aug 5;8(8):e70233. doi: 10.1371/journal.pone.0070233 (PMC3734070; doi:10.1371/journal.pone.0070233)
Supplement: Table S2 — Normalized Δ2-Ct values for the signature genes across all of the cell lines. (DOCX) [file pone.0070233.s008.docx]

**Table S2.** Normalized Δ^2^-Ct values for the signature genes across all of the cell lines.

| **Gene** | **Ager** | **Cdh5** | **Abcc4** | **Cldn12** | **Abcg2** | **Abcb1b** | **Lrp1** | **Slc7a1** | **Cldn5** | **Slc2a1** | **Bsg** | **Plvap** | **Cldn3** | **Gpr126** |
| --- | --- | --- | --- | --- | --- | --- | --- | --- | --- | --- | --- | --- | --- | --- |
| bEnd5-ctrlCM1 | 1.72 | 0.82 | 1.37 | 1.95 | 0.53 | 1.04 | 17.47 | 4.80 | 0.98 | 0.23 | 0.88 | 0.01 | 0.22 | 1.02 |
| bEnd5-WntCM1 | 1.27 | 0.83 | 1.30 | 2.21 | 0.57 | 1.08 | 14.76 | 6.08 | 1.34 | 0.24 | 0.88 | 0.02 | 0.71 | 0.94 |
| H5V-ctrlCM | 2.70 | 0.65 | 0.49 | 0.70 | 0.61 | 0.36 | 1.45 | 1.71 | 0.00 | 0.27 | 0.94 | 0.02 | 0.03 | 1.59 |
| H5V-WntCM | 2.36 | 0.62 | 0.55 | 0.85 | 0.73 | 0.29 | 1.32 | 1.92 | 0.00 | 0.40 | 0.95 | 0.02 | 0.14 | 1.25 |
| Lung-ctrlCM | 2.98 | 0.62 | 0.40 | 0.26 | 0.21 | 0.05 | 0.05 | 0.39 | 0.01 | 0.06 | 0.60 | 0.07 | 0.01 | 0.22 |
| Lung-WntCM | 2.25 | 0.56 | 0.53 | 0.28 | 0.28 | 0.05 | 0.03 | 0.44 | 0.01 | 0.07 | 0.57 | 0.07 | 0.05 | 0.33 |
| bEnd5-ctrlCM2 | 1.50 | 0.72 | 0.59 | 1.30 | 0.49 | 0.46 | 12.98 | 3.48 | 1.16 | 0.29 | 1.13 | 0.02 | 0.28 | 0.31 |
| bEnd5-WntCM2 | 1.12 | 0.62 | 0.68 | 1.20 | 0.48 | 0.44 | 7.47 | 3.01 | 1.72 | 0.27 | 1.15 | 0.03 | 0.46 | 0.32 |
| bEnd5-ctrlCM3 | 1.66 | 0.37 | 0.65 | 0.50 | 0.35 | 0.24 | 2.83 | 0.95 | 0.76 | 0.12 | 0.31 | 0.01 | 0.31 | 0.24 |
| bEnd5-WntCM3 | 1.69 | 0.52 | 0.93 | 0.51 | 0.46 | 0.34 | 1.96 | 1.18 | 1.43 | 0.15 | 0.36 | 0.02 | 0.15 | 0.25 |
| bEnd5-DMSO | 1.78 | 0.82 | 0.58 | 0.54 | 0.63 | 0.30 | 0.65 | 2.22 | 1.44 | 0.07 | 0.44 | 0.02 | 0.07 | 0.10 |
| bEnd5-BIO | 1.94 | 0.57 | 0.68 | 0.44 | 0.69 | 0.25 | 0.68 | 1.75 | 1.58 | 0.08 | 0.55 | 0.03 | 0.07 | 0.16 |
| bEnd5-6-BIO | 1.29 | 0.63 | 0.47 | 0.54 | 0.39 | 0.27 | 3.05 | 2.93 | 1.09 | 0.07 | 0.79 | 0.02 | 0.31 | 0.20 |
| bEnd5-PBS | 1.07 | 0.70 | 0.45 | 0.63 | 0.42 | 0.37 | 3.52 | 4.27 | 1.25 | 0.07 | 0.45 | 0.02 | 0.34 | 0.15 |
| bEnd5-WntPr | 1.17 | 0.92 | 0.64 | 0.68 | 0.59 | 0.50 | 5.36 | 4.88 | 1.40 | 0.08 | 0.52 | 0.02 | 0.29 | 0.19 |
| bEnd5-WntRD | 0.80 | 0.34 | 0.42 | 0.33 | 0.37 | 0.26 | 1.85 | 1.19 | 1.35 | 0.05 | 0.37 | 0.04 | 0.16 | 0.15 |
| bEnd5-GFP | 1.75 | 0.53 | 0.62 | 0.47 | 0.58 | 0.37 | 0.13 | 1.26 | 1.44 | 0.08 | 0.39 | 0.02 | 0.11 | 0.15 |
| bEnd5-β-CTA | 1.59 | 0.76 | 0.68 | 1.74 | 0.41 | 0.46 | 10.65 | 3.74 | 0.44 | 0.35 | 1.14 | 0.08 | 1.41 | 0.31 |
| +Astros | 0.77 | 0.50 | 1.08 | 0.75 | 0.73 | 0.22 | 0.13 | 2.49 | 1.22 | 0.26 | 1.56 | 0.04 | 0.27 | 1.62 |

Abbreviations: **bEnd5-ctrlCM** or **WntCM 1**: activation with undiluted controlCM or Wnt 3aCM for 3 days; **2**: undiluted controlCM or Wnt3aCM for 24 hours; **3**: diluted (1 to 3) controlCM or Wnt3aCM for 24 hours. **H5V** or **Lung-ctrlCM** or **-Wnt3aCM**: activation with undiluted controlCM or Wnt3aCM for 3 days. **bEnd5-DMSO**, **-BIO** or **-6-BIO**: activation with DMSO, BIO or 6-BIO for 24 hours. **bEnd5-PBS** or **-WntPr** or **-WntRD**: activation with PBS, recombinant Wnt3a from Peprotech or R&D systems for 24 hours. **bEnd5-GFP** or **-**β**-CTA**: bEnd5 cells infected with lenti-GFP or lenti-LEFΔN-β-CTA. **+Astros**: co-cultures of bEnd5 with astrocytes.
